# Efficacy and safety of immune checkpoint inhibitor monotherapy in elderly patients with non‐small cell lung cancer

**DOI:** 10.1002/agm2.12147

**Published:** 2021-01-29

**Authors:** Yasuhiro Chikaishi, Masaaki Inoue, Kasumi Kusanagi, Yohei Honda, Junichi Yoshida, Masao Tanaka

**Affiliations:** ^1^ Department of Chest Surgery Shimonoseki City Hospital Shimonoseki Japan

**Keywords:** elderly patient, immune checkpoint inhibitor, non‐small cell lung cancer

## Abstract

The efficacy and safety of immune checkpoint inhibitor (ICI) monotherapy in elderly patients with non‐small cell lung cancer (NSCLC) remain unclear, especially in patients older than 80 years. We retrospectively reviewed the records of 10 patients older than 80 years with NSCLCs treated by ICIs. The median age was 85 years (range, 82‐93 years), and 7 patients were men. The median length of follow‐up was 13 months (range, 4.5‐23 months). Eight patients had adenocarcinoma (3 of whom had exon 19 deletions), and two had squamous cell carcinoma. Expression of programmed cell death ligand 1 (PD‐L1) was ≥ 50% in 3 patients, between 1% and 49% in 4 patients, < 1% in 1 patient, and undetected in 2 patients. Patients with undetected PD‐L1 underwent transbronchial lung biopsy. Performance status was graded zero, one, and two in two, seven, and one patients, respectively. First‐, second‐, and third‐line treatments were administered to three, three, and four patients, respectively. The 2‐year overall survival rate was 30.0% (median, 285 days). Time to treatment failure rate on the 2 years was 10.0% (median, 167 days). One patient achieved a partial response, and one achieved a complete response. ICI‐associated adverse events occurred in five patients. In summary, ICIs were effective in some patients older than 80 years; however, some experienced adverse effects. Elderly patients must be selected carefully for ICI treatment.

**Key Point**

**Significant findings of this study**

We evaluated the efficacy and safety of immune checkpoint inhibitor (ICI) monotherapy in elderly patients with non‐small cell lung cancer.

**What this study adds**

In some patients older than 80 years, ICIs were effective, but others experienced adverse effects. Patients older than 80 years must be selected carefully for ICI treatment.

## 1 INTRODUCTION

Immunotherapy directed against programmed cell death protein 1 signaling has produced good responses in patients with advanced non‐small cell carcinoma (NSCLC).^1^, ^2^, ^3^, ^4^, ^5^ Immune checkpoint inhibitors (ICIs) have been used to treat NSCLC, and even chemotherapy‐resistant patients have shown good results.^1^, ^2^ Nivolumab, which was the first ICI approved for chemotherapy‐resistant patients, produced better rates of progression‐free survival than did standard second‐line chemotherapy in a phase III clinical trial.^1^, ^2^ Three ICIs have been approved for NSCLC as monotherapy in Japan: nivolumab, pembrolizumab, and atezolizumab. Pembrolizumab is approved as first‐line chemotherapy for patients with positive expression of programmed cell death ligand 1 (PD‐L1).^3^, ^6^ Nivolumab and atezolizumab are approved for chemotherapy‐resistant patients. Moreover, for patients with untreated NSCLC, platinum‐based doublet combinations with ICI therapy have resulted in better progression‐free survival and overall survival.^7^, ^8^

Several studies have shown that certain cytotoxic agents are safe and effective in elderly patients.^9^, ^10^, ^11^, ^12^ Standard chemotherapy for elderly patients is docetaxel monotherapy or carboplatin plus pemetrexed.^11^, ^12^ However, the safety and effectiveness of ICIs in patients with NSCLC who are elderly or have poor performance status remain unclear. It is important to investigate the safety and effectiveness of ICIs.

Herein, we present data from patients older than 80 years in whom NSCLC was treated by ICIs at a single institution.

## 2 PATIENTS AND METHODS

We retrospectively examined data from patients older than 80 years who received ICIs at our institution from December 2015 to April 2019 for NSCLC, regardless of treatment duration. The population included patients with advanced and recurrent disease.

Baseline clinical characteristics were determined by retrospective review of medical records at the beginning of ICI administration.

Specimens for diagnosis were obtained after surgery, transbronchial biopsy, computed tomography‐guided biopsy, and pleural effusion.

Patients were administered the full ICI dose in each treatment session. Patients continued treatment until the emergence of progressive disease, development of unacceptable toxic effects, or withdrawal of consent to treatment. When ICI treatment was suspended, some patients then selected systemic chemotherapy, and others selected best supportive care.

We assessed overall survival, time to treatment failure after initial ICI administration, and the detailed treatment course of each patient. Overall survival was determined from the date of ICI initiation to the date of death from any cause or the follow‐up during survival. Time to treatment failure was measured from the first day of initial ICI intake to the date of discontinuance for any reason or the last follow‐up during survival. We set November 2020 as the time of data cutoff. Tumor responses were classified as complete responses, partial responses, stable disease, or progressive disease, according to the Response Evaluation Criteria in Solid Tumors guidelines.^13^

The Kaplan–Meier method was used to calculate survival curves. Statview version 5.0 (Abacus Concepts, Inc.) was used for all statistical analyses.

## 3 RESULTS

### 3.1 Patients

From December 2015 to April 2019, 10 patients older than 80 years received ICIs for NSCLC at our institution. Table 1 summarizes the patients’ characteristics at baseline, initiation of ICI administration, and patients’ demographic and clinical characteristics. Detailed descriptions are provided in Table 2.

**TABLE 1:** Baseline clinical and pathological characteristics of the enrolled patients

Clinical factor	Category	No. of patients
Gender	Female/male	3/7
Age, y	Median/average	85/86
	Range	82‐93
Smoking status	Current smoker	2
	Never smoker	2
	Former smoker	3
	Unknown	3
PS (ECOG)	0/1/2	2/7/1
Histology	Adenocarcinoma (19del)	8 (3)
	Squamous	2
PD‐L1	Over 50%	3
	1%‐49%	3
	Under 1%	2
	Undecidable	2
Treatment history	Yes	7
	Second line/over third line	3/4
	No	3
Stage	III/IV/recurrence	1/7/2
Initial ICIs	Nivolumab	2
	Pembrolizumab	6
	Atezolizumab	2

Abbreviations: ECOG, Eastern Cooperative Oncology Group; ICIs, immune checkpoint inhibitors; PD‐L1, programmed cell death ligand 1; PS, Performance Status

**TABLE 2:** Baseline clinical and pathological characteristics of the enrolled each patient

Case	Age, y/sex	PS	Smoking status	Histology	PD‐L1, %	Timing of ICI	Specimens for diagnosis	Stage	Initial ICIs
1	82/F	1	Never	Adenoca.	50‐60	7th	Surgery	Recurrence	Pembrolizumab
2	93/M	1	Unknown	Adenoca.	10	2nd	Surgery	IVA	Pembrolizumab
3	85/M	1	Current	Adenoca.	1‐24	6th	Pleural effusion	IVA	Nivolumab
4	82/M	1	Unknown	Sq.	< 1	2nd	Surgery	Recurrence	Atezolizumab
5	84/M	1	Current	Adenoca.	Undecidable	3rd	TBB	IV A	Nivolumab
6	89/F	0	Unknown	Adenoca.	75	1st	Surgery	IV A	Pembrolizumab
7	88/M	1	Former	Adenoca.	1	3rd	Surgery	IV B	Pembrolizumab
8	82/M	1	Unknown	Adenoca.	90	1st	TBB	IV B	Pembrolizumab
9	84/M	0	Former	Sq.	1 ~ 24	1st	TBB	III B	Pembrolizumab
10	91/F	2	Former	Adenoca.	Undecidable	2nd	CT guided biopsy	IVA	Atezolizumab

Abbreviations: Adenoca., adenocarcinoma; CT, computed tomography; F, female; ICI, immune checkpoint inhibitor; M, male; PD‐L1, programmed cell death ligand 1; PS, performance status; Sq., squamous cell carcinoma; TBB, transbronchial lung biopsy.

The median age was 85 years (range, 82‐93 years); 2 patients were older than 90 years. Performance status was rated zero for two patients, one for seven patients, and two for one patient. Seven patients (70%) were men; eight patients had a diagnosis of adenocarcinoma (3 of whom had exon 19 deletions), and two had a diagnosis of squamous cell carcinoma. Before ICI treatment, seven patients had undergone at least first‐line treatment for NSCLC. At initiation of ICI treatment, two patients had exhibited recurrent disease postoperatively, one patient had stage III disease, and seven patients had stage IV disease. For initial ICI treatment, two patients received nivolumab, six received pembrolizumab, and two received atezolizumab.

### 3.2 Efficacy

**FIGURE 1:**
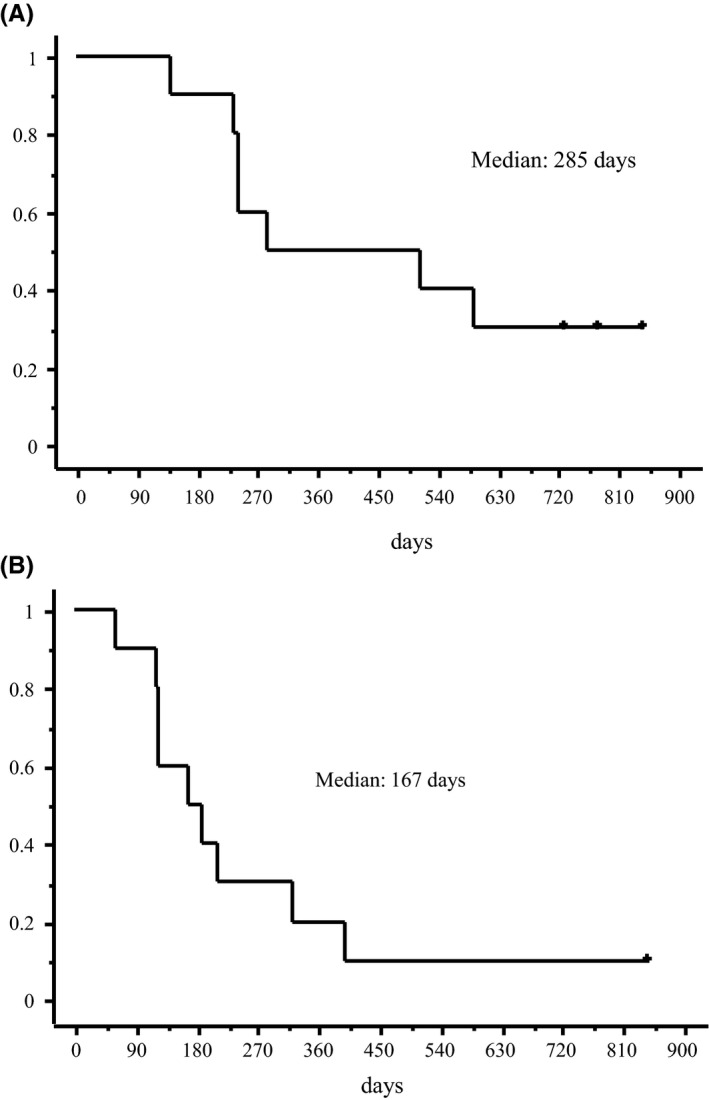
Kaplan–Meier curves for all patients who received immune checkpoint inhibitor (ICI) monotherapy for non‐small cell lung cancer. A, Overall survival. B, Time to treatment failure

**FIGURE 2:**
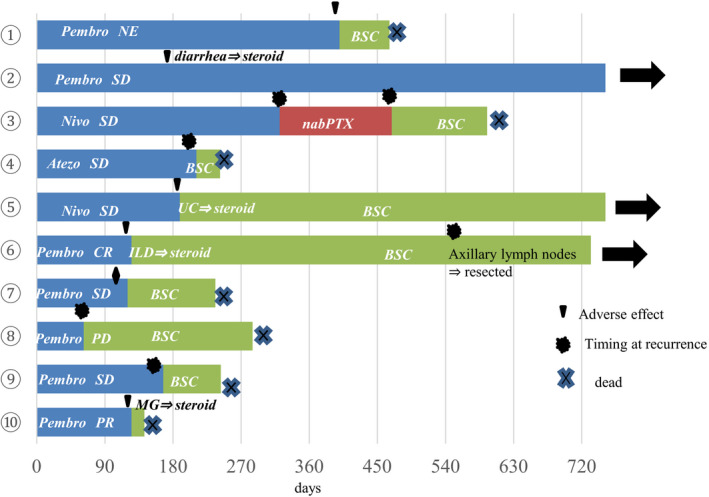
Individual swimmer pilots for all patients (denoted by numbers in circles) who received immune checkpoint inhibitor (ICI) monotherapy for non‐small cell lung cancer (0 represents the beginning of ICI treatment). Black arrows indicate patients who were alive at the time of data cutoff. Atezo, atezolizumab; BSC, best supportive care; CR, complete response; ILD, interstitial lung disease; MG, myasthenia gravis; nabPTX, nab‐paclitaxel; NE, not evaluated; Nivo, nivolumab; PD, progressive disease; Pembro, pembrolizumab; PR, partial response; SD, stable disease; UC, ulcerative colitis

At the time of data cutoff, the median length of follow‐up was 13 months (range, 4.5‐28 months). Among the 10 patients, the 2‐year overall survival rate was 30.0% (Figure 1A). Time to treatment failure rate on the 2 years was 10.0% (Figure 1B). Initial treatment outcomes for all patients administered ICIs are depicted in Figure 2 as swimmer plots. One patient achieved a partial response, and one achieved a complete response. Adverse events associated with ICIs occurred in five patients: ulcerative colitis in three, interstitial lung disease in one, and myasthenia gravis in one. Three of the five patients were treated with steroids. In 9 patients, ICIs were administered over a 4‐month period. Treatment lasted longest for one patient (case 2 in Table 2), in whom stable disease was achieved. In this case, the patient complained of diarrhea (Common Terminology Criteria for Adverse Events [CTCAE] version 5.0,^14^ grade 2). Pembrolizumab was discontinued, and methylprednisolone (500 mg/body) was administered for 3 days. Pembrolizumab treatment resume after methylprednisolone treatment. Two patients (cases 5 and 6 in Table 2) achieved long survival after ICI treatment was discontinued because of immune‐related adverse events (irAEs). In case 5, the patient complained of diarrhea (CTCAE grade 4), ulcerative colitis was diagnosed by colonoscopy, and methylprednisolone treatment (1 mg/kg/day) was started. Methylprednisolone treatment was administered for 2 months with tapering. In case 6, the tumor response was a complete response; however, interstitial lung disease occurred. Methylprednisolone treatment (1 mg/kg/day) was started; maintenance treatment (5‐10 mg/body) was necessary for approximately 8 months in this patient.

## 4 DISCUSSION

To the best of our knowledge, this is the only retrospective study of ICI treatment in patients older than 80 years in a real‐world single institution setting; in some reports, patients have been 75 years of age or older.^15^, ^16^ Moreover, there are few retrospective studies from the Department of Chest Surgery of the single institution.

In our study, 9 of 10 patients received ICIs over a 4‐month period. It seems controversial whether these results should be considered long or short term, but other studies of ICI have demonstrated progression‐free survival for < 4 months.^1^, ^2^ Because our study included patients with progressive disease, it is not possible to conclude that our data reflected similar treatment outcomes.

Of our patients, one (case 2) received treatment for more than 2 years, and two (cases 5 and 6) are still alive more than 2 years since ICI treatment, despite the need to discontinue treatment because of irAEs. According to some reports,^1^, ^2^, ^3^, ^4^ ICIs can achieve long‐term survival (the so‐called tail plateau^17^). As in our older patients, ICIs may be administered safely and effectively in selected cases.

In this study, 5 of the 10 patients experienced irAEs more severe than CTCAE grade 2. Three patients received steroid treatment for irAEs; all three experienced improvement in irAEs. Major clinical studies^1^, ^2^, ^3^, ^4^ have shown that irAEs of any grade occur in 19.4%‐69% of patients receiving chemotherapy, and 0.8%‐15% experience irAEs of CTCAE grades 3‐5. In this study, 50% of our patients experienced irAEs of any grade, and in 20%, irAEs were graded higher than 3. The rate of irAEs is slightly worse in this study than in previous studies. Because this study focused on older patients, however, ICI may not have been the cause of irAE. In particular, two patients who experienced diarrhea did not undergo colonoscopy because of their age; thus, they may have had diverticulitis or other conditions. The irAEs caused by ICIs were serious conditions (ulcerative colitis, myasthenia gravis, and interstitial lung disease); thus, irAEs that develop during ICI therapy must be investigated and treated accordingly.

The present study has an important limitation that it was a retrospective study and only had 10 patient’s data. We believe our real‐world data are nonetheless important. This study has used data from a single institution. However, the retrospective study of 10 patients older than 80 years who received ICIs for NSCLC at a single institution has been rarely reported. Moreover, our study includes 2 patients older than 90 years.

## 5 CONCLUSIONS

ICI treatment may be effective in patients older than 80 years. ICIs may be key drugs for elderly patients with NSCLC if major irAEs can be managed without curtailing activities of daily living. However, in some cases, severe adverse effects occurred and had to be managed with steroids. Patients older than 80 years must be selected carefully for ICI treatment.

## CONFLICTS OF INTEREST

Nothing to disclose.

## AUTHOR CONTRIBUTIONS

*Writing of the paper:* Chikaishi. *Design of the study:* Chikaishi and Inoue. *Review of the study:* Chikaishi. *Data collection:* Chikaishi, Kusanagi, Honda, and Yoshida. *Review of medical records:* Chikaishi, Kusanagi, Honda, and Yoshida. *Statistical analysis:* Chikaishi. *Literature review:* Inoue. *Read and approved the final manuscript:* Inoue and Tanaka. *Initial statistical analysis:* Yoshida.

## References

[1] Brahmer J , Reckamp KL , Baas P , et al. Nivolumab versus docetaxel in advanced squamous‐cell non‐small‐cell lung cancer. N Engl J Med. 2015;9(2):123‐135. 10.1056/NEJMoa1504627 PMC4681400 26028407

[2] Borghaei H , Paz‐Ares L , Horn L , et al. Nivolumab versus docetaxel in advanced nonsquamous non‐small‐cell lung cancer. N N Engl J Med. 2015;22(17):1627‐1639. 10.1056/NEJMoa1507643 PMC5705936 26412456

[3] Garon EB , Rizvi NA , Hui R , et al. Pembrolizumab for the treatment of non‐small‐cell lung cancer. N Engl J Med. 2015;21(21):2018‐2028. 10.1056/NEJMoa1501824 25891174

[4] Rittmeyer A , Barlesi F , Waterkamp D , et al. Atezolizumab versus docetaxel in patients with previously treated non‐small‐cell lung cancer (OAK): a phase 3, open‐label, multicentre randomised controlled trial. Lancet. 2017;21(10066):255‐265. 10.1016/S0140-6736(16)32517-X PMC6886121 27979383

[5] Ichiki Y , Taira A , Chikaishi Y , et al. Prognostic factors of advanced or postoperative recurrent non‐small cell lung cancer targeted with immune check point inhibitors. J Thorac Dis. 2019;11(4):1117‐1123. 31179053 10.21037/jtd.2019.04.41 PMC6531735

[6] Reck M , Rodríguez‐Abreu D , Robinson AG , et al. Pembrolizumab versus chemotherapy for PD‐L1‐positive non‐small‐cell lung cancer. N Engl J Med. 2016;10(19):1823‐1833. 10.1056/NEJMoa1606774 27718847

[7] Gandhi L , Rodríguez‐Abreu D , et al. Pembrolizumab plus chemotherapy in metastatic non‐small‐cell lung cancer. N Engl J Med. 2018;31(22):2078‐2092. 10.1056/NEJMoa1801005 29658856

[8] Socinski MA , Jotte RM , Cappuzzo F , et al. Atezolizumab for first‐line treatment of metastatic nonsquamous NSCLC. N Engl J Med. 2018;14(24):2288‐2301. 10.1056/NEJMoa1716948 29863955

[9] Hesketh PJ , Lilenbaum RC , Chansky K , et al. Chemotherapy in patients > or = 80 with advanced non‐small cell lung cancer: combined results from SWOG 0027 and LUN 6. J Thorac Oncol. 2007;2(6):494‐498. 17545843 10.1097/JTO.0b013e318060097e

[10] Abe T , Takeda K , Ohe Y , et al. Randomized phase III trial comparing weekly docetaxel plus cisplatin versus docetaxel monotherapy every 3 weeks in elderly patients with advanced non‐small‐cell lung cancer: the intergroup trial JCOG0803/WJOG4307L. J Clin Oncol. 2015;20(6):575‐581. 10.1200/JCO.2014.55.8627 25584004

[11] Quoix E , Zalcman G , Oster JP , et al. Carboplatin and weekly paclitaxel doublet chemotherapy compared with monotherapy in elderly patients with advanced non‐small‐cell lung cancer: IFCT‐0501 randomised, phase 3 trial. Lancet. 2011;17(9796):1079‐1088. 10.1016/S0140-6736(11)60780-0 21831418

[12] Okamoto I , Nokihara H , Yoh K , et al. Randomized phase III study comparing carboplatin plus pemetrexed followed by pemetrexed versus docetaxel in elderly patients with advanced non‐squamous non‐small‐cell lung cancer (JCOG1210/WJOG7813L). J Clin Oncol. 2019;37(15_suppl):9031.

[13] Eisenhauer EA , Therasse P , Bogaerts J , et al. New response evaluation criteria in solid tumours: revised RECIST guideline (version 1.1). J Eur J Cancer. 2009;45:228‐247. 10.1016/j.ejca.2008.10.026 19097774

[14] NIH . Common Terminology Criteria for Adverse Events (CTCAE); 2017. https://ctep.cancer.gov/protocoldevelopment/electronic_applications/docs/CTCAE_v5_Quick_Reference_8.5x11.pdf

[15] Yamaguchi O , Imai H , Minemura H , et al. Efficacy and safety of immune checkpoint inhibitor monotherapy in pretreated elderly patients with non‐small cell lung cancer. Cancer Chemother Pharmacol. 2020;85(4):761‐7771. 32193618 10.1007/s00280-020-04055-7

[16] Imai H , Wasamoto S , Yamaguchi O , et al. Efficacy and safety of first‐line pembrolizumab monotherapy in elderly patients (aged ≥ 75 years) with non‐small cell lung cancer. J Cancer Res Clin Oncol. 2020;146(2):457‐466. 31853661 10.1007/s00432-019-03072-1 PMC11804284

[17] Kataoka N , Kunimatsu Y , Tachibana Y , et al. Atezolizumab in combination with carboplatin and etoposide for heavily treated small cell lung cancer. Thorac Cancer. 2020;11(9):2740‐2742. 32706170 10.1111/1759-7714.13588 PMC7471014

